# Efficacy, retention, and safety of tofacitinib in real-life: Hur-bio monocentric experience

**DOI:** 10.3906/sag-2007-123

**Published:** 2021-02-26

**Authors:** Emre BİLGİN, Furkan CEYLAN, Emine DURAN, Bayram FARİSOĞULLARI, Ertuğrul Çağrı BÖLEK, Gözde Kübra YARDIMCI, Levent KILIÇ, Ali AKDOĞAN, Ömer KARADAĞ, Şule Apraş BİLGEN, Sedat KİRAZ, Ali İhsan ERTENLİ, Umut KALYONCU

**Affiliations:** 1 Division of Rheumatology, Department of Internal Medicine, Medical School of Hacettepe University, Ankara Turkey; 2 Department of Internal Medicine, Medical School of Hacettepe University, Ankara Turkey

**Keywords:** Rheumatoid arthritis, tofacitinib, real-life, predictor

## Abstract

**Background/aim:**

To assess the real-life efficacy, retention rate, and safety data of tofacitinib in rheumatoid arthritis (RA) patients.

**Materials and methods:**

We analyzed all patients registered in the HURBİO database who received at least 1 dose of tofacitinib. Patients who received at least one dose were included in retention analysis; patients with at least 1 control visit were included in efficacy and safety analysis. Factors predicting good response at the last follow-up visit were analyzed by logistic regression analysis. Drug retention rates were calculated using the Kaplan–Meier method and predictors of drug retention were determined by Cox proportional hazard model. Adverse events, reasons for switching, and discontinuation were also determined.

**Results:**

Two hundred and forty-seven (210, 85.0% female) patients were included in the study. The median duration of tofacitinib treatment was 10.2 (20.2) [med, (IQR)] months. Two hundred and four (82.6%) patients were included in safety and efficacy analysis; 45.6% of patients were in low-disease activity (LDA) state (DAS28-CRP ≤ 3.2). Predictors of LDA were being biologic-naïve [aOR 2.53 (1.31–4.88); 95% CI] and RF negativity [aOR 2.14 (1.12–4.07); 95% CI]. At 1 year, the overall tofacitinib retention rate was 63.9% with no relevant predicting factor. Response and retention rates of tofacitinib were similar in patients with and without concomitant csDMARDs. Treatment failure was the most common cause of discontinuation. The most common infectious and laboratory adverse events were herpes zoster infection (3.9 per 100 patient-years) and elevation in ALT (x3UNL: 9.7 per 100 patient-years), respectively.

**Conclusion:**

Tofacitinib is effective as monotherapy or in combination with csDMARDs. It is a well-tolerated treatment option in Turkish RA patients.

## 1. Introduction

Rheumatoid arthritis (RA) is a chronic immune-mediated disease characterized by systemic inflammation causing articular and extraarticular manifestations. Systemic inflammation is directly correlated with active disease and ongoing active disease may cause functional impairment, reduced quality of life, organ-system dysfunction, and even death [1]. The main principle of RA treatment is to reach sustained remission or low disease activity (LDA) in every patient [2]. 

In the last 20 years, biologic agents redesigned the principles of RA management. Despite the growing number of “biologic players”, there is still an unmet need in RA management. Approximately half of the patients do not respond sufficiently to conventional synthetic (cs) or biologic (b) disease-modifying antirheumatic drugs (DMARD), revealing the need for alternative treatments [3].

Tofacitinib is an oral pan-Janus kinase (JAK) inhibitor. Phase II and III clinical trials revealed the efficacy of tofacitinib, either as a monotherapy or in combination with csDMARDs, in RA patients [4–7]. Comparative studies of tofacitinib and other bDMARDs resulted in similar efficacy and safety profiles [4,7–10]. Although the safety and efficacy have been evaluated in clinical trials, there is still a need for the real-life experience of tofacitinib to confirm its role in RA management. 

In this study, our primary aim was to determine the real-life efficacy, retention rate, and safety profile of tofacitinib in RA patients treated at our center.

## 2. Methods

### 2.1. Study population

We conducted this retrospective longitudinal analysis with RA patients who received at least 1 dose of tofacitinib from March 2015 until the end of November 2019 and were registered in the Hacettepe University biological database (HUR-BİO) which was established in a 2005 study [11]. The diagnosis of RA was established by a treating physician with taking into account the history, physical examination, laboratory, and imaging of the patients. All patients met the 1987 American College of Rheumatology (ACR) and/or 2010 European League Against Rheumatism (EULAR)/ACR classification criteria [12,13].

According to Turkish Social Security and Prescription rules, patients receiving biologic/targeted-synthetic DMARDs ought to be seen every 3 months by the treating physician. With the aid of these regulations, many patients are in regular follow-up and we could identify whether the patients actually received the drug. If the patient had no control visit for 6 months after prescription, the treating physician made a constructed phone call with the patient or their relatives to confirm whether the patient received tofacitinib. A total of 275 RA patients were prescribed tofacitinib; 28 (10.2%) of them never received the drug. As a result, our main study population consisted of 247 patients who received at least 1 dose of tofacitinib.

### 2.2. Data collection 

#### 2.2.1. Demographic data and population characteristics

We collected the following demographic data: sex, age, smoking history, body mass index (BMI), frequency of hypertension and diabetes mellitus. Regarding RA, disease duration rheumatoid factor (RF) and anticyclic citrullinated peptide (anti-CCP) positivity, duration under tofacitinib, percentage of biologic-naïve patients, distribution of previous bDMARDs in the biologic-experienced group, concomitant DMARD [methotrexate (MTX), leflunomide (LEF), sulphasalazine (SLZ), hydroxychloroquine (HCQ)] and glucocorticoid (GC) use at last visit, baseline disease activity and functional status parameters [erythrocyte sedimentation rate (ESR) (mm/h), C-reactive protein (CRP) (mg/dL), tender and swollen joint count (28 joints), patient global-visual analog scale assessment (VAS) (0–100 mm) (PGA-VAS), disease activity score (DAS) 28-ESR, and health assessment questionnaire-disability index (HAQ-DI)] were recorded.

For the main analyses, we grouped patients as biologic-naïve vs. biologic-experienced and tofacitinib monotherapy vs. tofacitinib + concomitant csDMARDs.

#### 2.2.2. Assessment of efficacy

Patients who had at least 1 control visit under tofacitinib and complete baseline disease activity data were included in the efficacy analysis. To test the overall effectiveness of tofacitinib, we compared the ESR, CRP, PGA-VAS, tender and swollen joint counts, DAS28, and HAQ-DI scores at the visit just before starting tofacitinib and the last visit of the patient under tofacitinib therapy. As physician global assessment has not been recorded in our database, we could not compare the clinical disease activity index (CDAI) or simple disease activity index (SDAI) scores. Also, we had no missing values of DAS28 at the last visits of patients, so we decided to take this time point for comparison instead of the 3rd or 6th month of therapy and adjust the final model for the duration of tofacitinib therapy. Patients were categorized into 4 groups according to DAS28 score at last follow-up visit: Remission (DAS28 ≤ 2.6), low (2.6–3.2), moderate (3.3–5.1), and high (>5.1) [14]. Patients were further grouped as responders or nonresponders according to DAS28 at the last follow-up visit; DAS28 ≤ 3.2: responders; DAS28 > 3.2: nonresponders. We also used the EULAR response criteria to assess the efficacy of tofacitinib [15]. In this assessment, patients were categorized into 3 groups: good response (DAS28 improvement regarding baseline > 1.2 and DAS28 at last visit ≤ 3.2), moderate response (DAS28 improvement regarding baseline > 1.2 and DAS28 at last visit > 3.2 or DAS28 improvement regarding baseline > 0.6 to ≤ 1.2 and DAS28 at last visit ≤ 5.1), and no response (DAS28 improvement regarding baseline ≤ 0.6, irrespective of DAS28 at last visit or DAS28 improvement regarding baseline > 0.6 to ≤ 1.2 and DAS28 at last visit > 5.1). Besides evaluating disease activity, HAQ-DI scores at first and last visits (calculated for patients with baseline HAQ-DI score > 0.5) were compared to determine the effects of tofacitinib on the functional status of patients. A minimal clinical difference of HAQ-DI score has been proposed as 0.22 (calculated for patients with baseline HAQ-DI score > 0.5), and functional remission has been defined as HAQ-DI ≤ 0.5 in earlier studies [16,17]. We defined the percentage of patients who met these definitions.

#### 2.2.3. Assessment of retention rate

Patients who had at least 1 dose of tofacitinib were included in the drug retention analysis. To calculate the drug retention more precisely, patients to whom tofacitinib was prescribed and who have not had a control visit in the following 6 months were assigned into the tofacitinib-continue group if they had not been prescribed another biologic treatment. If the patients have not had a control visit for over 6 months and they had not been prescribed tofacitinib by another institution, they were assigned into the tofacitinib-discontinue group.

#### 2.2.4. Tofacitinib discontinuation and adverse events

Tofacitinib discontinuation and adverse event analysis was done on patients who had at least 1 control visit under tofacitinib and complete baseline data. Discontinuation rates and causes of discontinuation were analyzed for biologic-naïve and experienced groups.

For safety reasons, adverse events attributable to tofacitinib (neutropenia (<1500/mm3), leukopenia (<4000/mm3), transaminitis [alanine aminotransferase (ALT) > 3 X UNL (upper normal limit, UNL = 40 IU/mL)], changes in lipid profile (calculated for patients who had baseline and follow-up values), herpes zoster (HZ) infection and infections other than HZ, hepatitis reactivation, tuberculosis, cancer) were analyzed. Adverse events other than laboratory abnormalities were calculated per 100 patient-years.

### 2.3. Statistical analysis

Statistical analysis was performed using the Statistical Package for the Social Sciences software (version 25.0; IBM Corporation, Armonk, NY, USA). The variables were investigated using visual (histogram, probability plots) and analytic methods (Kolmogorov–Smirnov, skewness, and kurtosis) to determine whether they are normally distributed or not. The data of descriptive analysis were expressed as either mean ± standard deviation (SD) or the median, interquartile range (IQR). Categorical variables were compared with the chi-square test or Fisher’s exact test where appropriate. Student’s t-test and Mann–Whitney U test were used to compare the normally and nonnormally distributed continuous data between the two groups, respectively. 

The univariate effects of age, sex, disease duration, smoking history, BMI, history of biologic treatment, RF and CCP positivity, baseline ESR-CRP levels, the status of concomitant DMARD, and glucocorticoid use identified with univariate analyzes (P < 0.20) were further entered into the logistic regression analysis to determine independent predictors of remission or low disease activity based on DAS28 at last follow-up visit. The same method was also used to determine independent predictors of good EULAR response at the last follow-up visit. Hosmer–Lemeshow goodness-of-fit statistics were used to assess model fit. 

Possible factors (same factors tested for remission or low disease activity) on tofacitinib retention were investigated using the log-rank test. Kaplan–Meier survival estimates were calculated. Possible factors identified with univariate analyses (P < 0.20) were further entered into the Cox regression analysis with backward selection to determine independent predictors of tofacitinib retention. Among correlated factors with similar effects on tofacitinib retention, only those with clinical significance were included. The proportional hazards assumption and model fit were assessed by means of residual (Schonfeld and Martingale) analysis.

Adverse events other than the lipid profile were estimated for 100 patient-years. A 5% type-I error level was used to infer statistical significance.

## 3. Results

### 3.1. Study population and patient characteristics

A total of 247 patients were included in the study. The mean age was 53.1 ± 12.6 years and 210 (85.0%) patients were female. The mean disease duration was 11.4 ± 8.0 years. The current smoking ratio was 25.5%. Hypertension, diabetes mellitus, and obesity (BMI > 30) were prevalent in 30.1%, 13.0%, 47.0% of patients, respectively. RF, antiCCP, RF and/or anti-CCP positivity rates were 66.7%, 65.2%, and 79.7%, respectively. The rate of concomitant synthetic DMARD and GC use and disease activity parameters at the first visit were similar between biologic-naïve and biologic-experienced groups (Table 1). 

**Table 1:** Demographic and baseline disease characteristics of all patients, comparison of these variables among biologic-naïve and experienced patients.

Variables*	All patients(n = 247)	Biologic-naïve(n = 137, 55.5%)	Biologic-experienced(n = 110, 44.5%)	P
Female	210 (85.0)	116 (84.7)	94 (85.5)	0.86
Age, years (mean ± SD)	53.1 ± 12.6	53.7 ± 12.9	52.3 ± 12.3	0.37
Disease duration, years (mean ± SD)	11.4 ± 8.0	9.5 ± 7.5	13.6 ± 8.0	<0.001
Smoking- Current smoker- Ex-smoker or never smoked	63 (25.5)184 (74.5)	31 (22.6)106 (77.4)	32 (29.1)78 (70.9)	0.24
BMI ≥ 30	116 (47.0)	63 (46.0)	53 (48.2)	0.73
Hypertension	74 (30.1)	38 (27.7)	36 (33.0)	0.36
Diabetes	32 (13.0)	17 (12.4)	15 (13.8)	0.75
Positive RF (n=240)	160 (66.7)	93 (69.4)	67 (63.2)	0.31
Positive CCP (n=207)	135 (65.2)	81 (71.1)	54 (58.1)	0.06
Positive RF or CCP (n=236)	188 (79.7)	111 (83.5)	77 (74.8)	0.10
Duration under Tofacitinib, months (med, IQR)	10.2 (20.2)	10.9 (19.8)	9.5 (16.5)	0.13
Monotherapy (±GC) (at last visit)	41 (16.6)	21 (15.3)	20 (18.2)	0.55
Glucocorticoids (at last visit)	182 (73.7)	106 (77.4)	76 (69.1)	0.15
Combination with at least one csDMARD (at last visit)- Methotrexate- Leflunomide- Sulphasalazine- Hydroxychloroquine	206 (83.4)61 (24.7)70 (28.9)9 (3.7)135 (55.8)	116 (84.7)35 (25.5)32 (23.4)4 (2.9)83 (60.6)	90 (81.8)26 (23.6)38 (34.5)5 (4.5)52 (47.3)	0.540.720.070.490.04
Disease activity (at first visit) (n=220)- ESR - CRP - Tender joint count- Swollen joint count- Patient VAS global- DAS28- HAQ	27 (28)1.3 (1.5)4 (7)2 (4)70 (30)4.7 ± 1.41.05 (1.15)	26 (27)1.2 (1.4)4 (6)2 (4)70 (30)4.6 ± 1.40.95 (1.10)	28 (30)1.3 (1.6)4 (7)2 (4)70 (30)4.6 ± 1.31.15 (1.05)	0.910.560.640.740.750.860.07

* n (%), if otherwise specified.BMI: body mass index, CCP: cyclic-citrulinated peptide, csDMARD: conventional synthetic disease modifying antirheumatic drugs, CRP: C-reactive protein, DAS28: disease activity score 28, ESR: erythrocyte sedimentation rate, GC: glucocorticoids, HAQ: health assessment questionnaire, IQR: interquartile range, RF: rheumatoid factor, TOFA: tofaacitinib, VAS: visual analogue scale.

Overall, 137 (55.5%) patients were bDMARD-naïve. In bDMARD-experienced group (n = 110, 44.5%), the number of previous bDMARDs was [med, (IQR)] 3 (2–4). Of 110 patients, 44 (40.0%) had only anti-TNF, 23 (20.9%) had only nonanti-TNF, and 43 (39.1%) had at least one anti-TNF and nonanti-TNF bDMARDs before tofacitinib. Distribution of former bDMARD therapies: 59 (53.6%) adalimumab, 51 (46.4%) etanercept, 45 (40.9%) abatacept, 41 (37.3%) tocilizumab, 27 (24.5%) certolizumab, 23 (20.9%) rituximab, 21 (19.1%) infliximab, and 15 (16.6%) golimumab.

### 3.2. Tofacitinib efficacy and retention rate

#### 3.2.1. Efficacy

**Figure 1:**
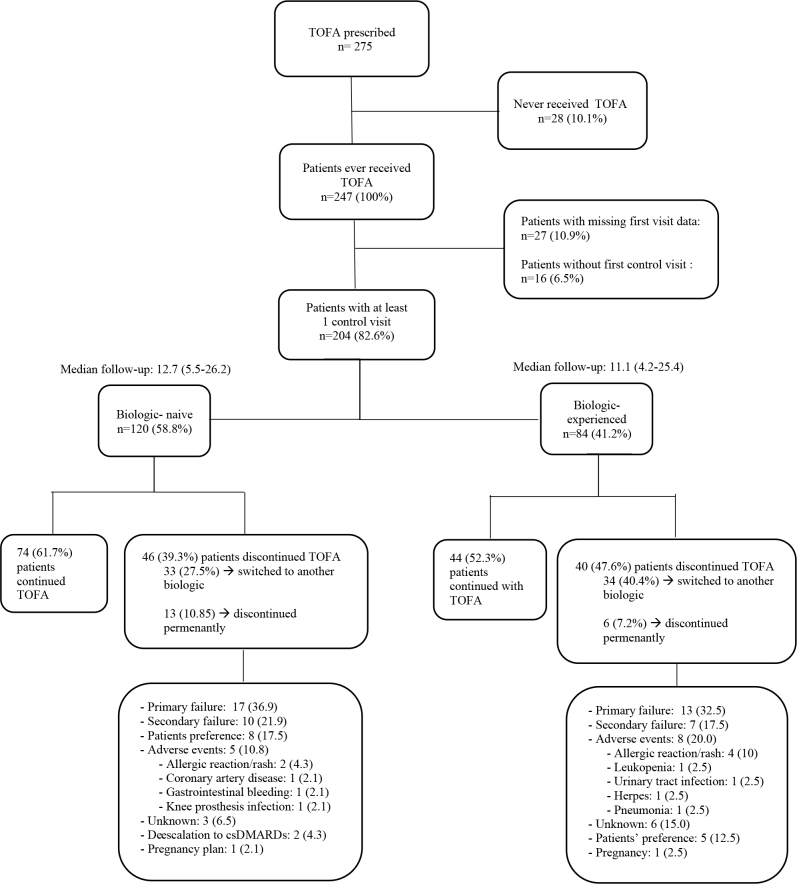
Flow chart of patient enrollment, causes of discontinuation.

Of 247 patients, 27 (10.9%) had missing first visit data and 16 (6.5%) did not have a first control visit yet by the date of data analysis. Patients with at least one control visit after starting tofacitinib and complete baseline data (204, 82.6%) were included in further analyses to compare the effectiveness of the drug (see Figure 1). The median follow-up of these patients when they have been receiving tofacitinib was 11.6 (20.7) [med, (IQR)] months. Baseline vs. last follow-up visit values for these parameters were as follows: ESR: 28 (29) vs. 22 (22); CRP: 1.2 (1.6) vs. 0.6 (0.8); SJC: 2 (4) vs. 0 (2); TJC: 5 (7) vs. 1 (5); PGA-VAS: 70 (30) vs. 50 (30); DAS28: 4.7 ± 1.4 vs. 3.6 ± 1.5; HAQ-DI: 1.02 (1.10) vs. 0.65 (1.01); P < 0.001 for all parameters. 

**Figure 2:**
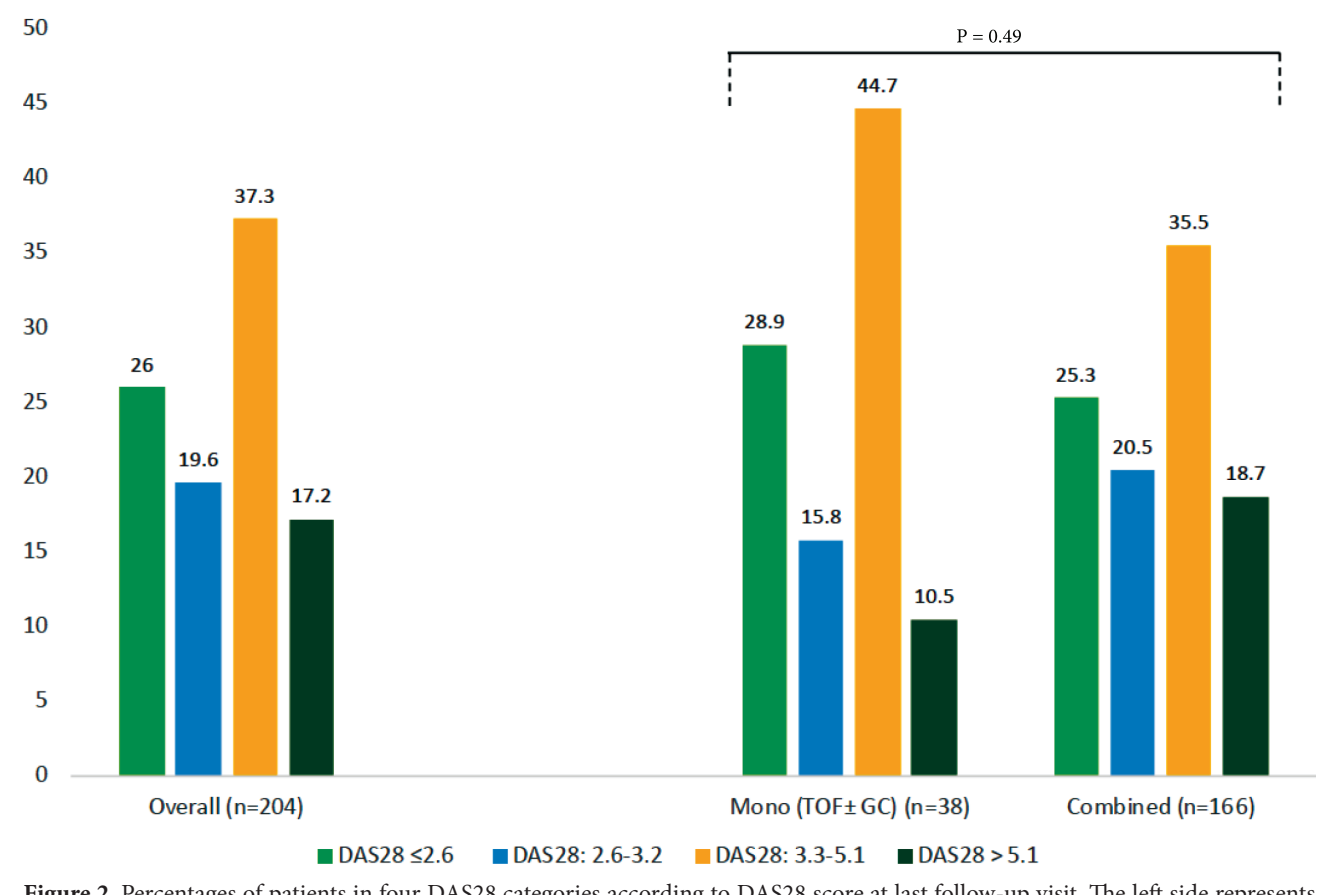
Percentages of patients in four DAS28 categories according to DAS28 score at last follow-up visit. The left side represents the overall group, the right side represents data according to concomitant csDMARD use at the last follow-up visit (tofacitinib ± glucocorticoids vs. tofacitinib + DMARDs ± glucocorticoids).

The distribution of patients into DAS28 categories (remission, low, moderate, high) was 26.0%, 19.6%, 37.3%, and 17.2%, respectively. The percentage of patients fitting into different disease activity categories according to their concomitant DMARD use [monotherapy (±GCs) vs. combination] were similar. The details of patients’ distribution are given in Figure 2. 

Overall, 45.6% of patients were in the “responders” group and 54.4% of patients were in the “nonresponders” group. Predictors of response (DAS28-CRP ≤ 3.2 at last follow-up visit) were determined by logistic regression analysis. Predictors of good response to tofacitinib were (in multivariate analysis, adjusted for follow-up duration under tofacitinib, RA disease duration, and baseline DAS28 score): being biologic-naïve [OR 2.53 (1.31–4.88); 95% CI] and RF negativity [OR 2.14 (1.12–4.07); 95% CI] (Table 2). Model fit was tested by the Hosmer–Lemeshow test (P = 0.46). Response (DAS28-CRP ≤ 3.2) rates were 53.3% vs. 34.5% in biologic-naïve and biologic-experienced patients, respectively; 56.9% vs. 40.4% in RF negative and RF positive patients, respectively. Anti-CCP status or seropositivity status (RF and/or anti-CCP positivity or absence of both) were statistically insignificant when they were entered into the model one by one instead of RF status. 

 According to EULAR response criteria, 45.6%, 22.1%, and 32.4% of patients met the good, moderate, and no response criteria, respectively, at the last follow-up visit. Predictors of good EULAR response criteria were (in multivariate analysis, adjusted for follow-up duration under tofacitinib, RA disease duration, and baseline DAS28 score): being biologic-naïve [OR 2.70 (1.40–5.25); 95% CI] and RF negativity [OR 2.17 (1.13–4.16); 95% CI]. Anti-CCP status or seropositivity status (RF and/or anti-CCP positivity or absence of both) were statistically insignificant when they entered into the model one by one instead of RF status. Model fit was tested by the Hosmer–Lemeshow test (P = 0.30).

**Table 2:** Predictors of good response* to tofacitinib.

	Univariate analysis		Multivariate analysis †,‡		Final multivariate model†,¶	
	Odds ratio (95% CI)	P value	Adjusted odds ratio(95% CI)	P value	Adjusted odds ratio(95% CI)	P value
Sex(female vs. male)	1.98 (0.90–4.36)	0.11	1.42 (0.57–3.58)	0.44		
Smoking (current vs. ex-never)	0.97 (0.51–1.84)	0.93				
BMI (>25 vs. <25)	1.27 (0.66–2.41)	0.46				
History of biologic treatment(naïve vs. experienced)	2.17 (1.22–3.85)	0.008	2.44 (1.22–4.87)	0.011	2.53 (1.31–4.88)	0.005
Rheumatoid factor (negative vs. positive)	1.94 (1.07–3.54)	0.03	1.89 (0.97–3.70)	0.062	2.14 (1.12–4.07)	0.021
Anti-CCP antibody (negative vs. positive)	1.41 (0.74–2.67)	0.29				
Baseline ESR(>20 mm/h vs. normal)	0.61 (0.33–1.12)	0.12	0.87 (0.42–1.83)	0.73		
Baseline C-reactive protein(>0.8 mg/dL vs. normal)	0.74 (0.41–1.35)	0.33				
Concomitant csDMARD (yes vs. no)	1.04 (0.51–2.11)	0.90				

*Good response means DAS28 ≤ 3.2 at last follow-up visit.†Adjusted for follow-up duration under tofacitinib, RA disease duration and baseline DAS28 score; ‡ Variables with P < 0.20 in univariate analyses were included. This is the baseline model.¶ Logistic regression with backward LR.

At the first visit, 26% of patients had a HAQ-DI score ≤ 0.5, while 45% of patients had a HAQ-DI score ≤ 0.5 at the last follow-up visit (P < 0.001). The mean difference of HAQ-DI scores and HAQ-DI drop ≥ 0.22 at the last follow-up visit compared to the first visit were calculated for 150 (74%) patients who had a baseline HAQ-DI score > 0.5. The mean difference was 0.40 (95% CI, 0.30–0.40, P < 0.001) and HAQ-DI decrease ≥ 0.22 was valid for 83/150 (55.3%) patients. 

#### 3.2.2. Retention

**Figure 3:**
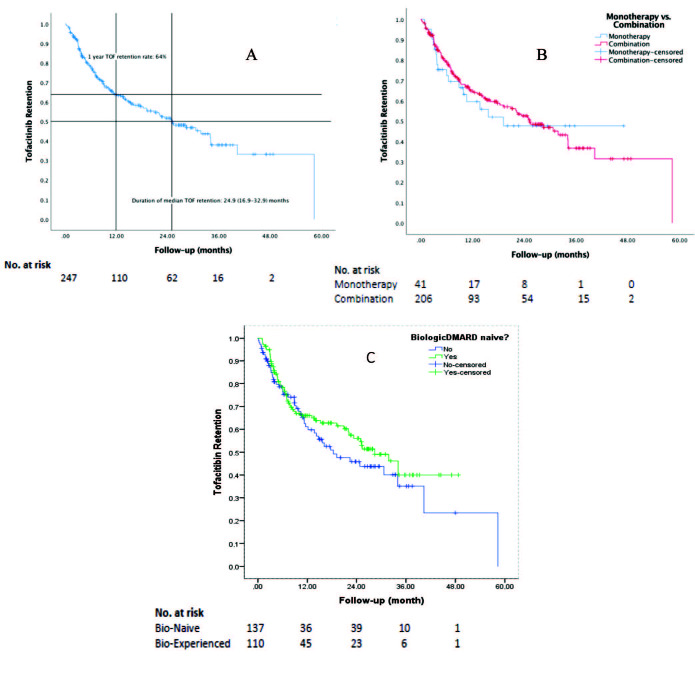
Retention analysis of tofacitinib (by Kaplan–Meier and log-rank comparison). A) Unadjusted tofacitinib retention in rheumatoid arthritis patients; B) Unadjusted tofacitinib retention according to concomitant csDMARD use (tofacitinib ± glucocorticoids vs. tofacitinib + DMARDs ± glucocorticoids); C) Unadjusted tofacitinib retention according to concomitant previous biologic DMARD use.

The tofacitinib retention rate was calculated over the whole study population (n = 247, 100%). The median duration of tofacitinib treatment was 10.2 (20.2) [med, (IQR)] months and similar among biologic-naïve and biologic-experienced groups. At 1 year, the overall tofacitinib retention rate was 63.9% (Figure 3A). The median tofacitinib retention was 24.9 (16) [med, (IQR)] months. Unadjusted tofacitinib retention rates were similar in patients receiving tofacitinib as monotherapy (±GCs) or combination with DMARDs (1-year retention: monotherapy (±GCs) vs. combination: 59.7% vs. 64.8%, P = 0.76, Figure 3B). Unadjusted tofacitinib retention rates were similar in bDMARD-naïve and experienced patients (1-year retention: bDMARD-naïve vs. bDMARD-experienced: 59.6% vs. 65.2%, P = 0.26, Figure 3C). In multivariate analysis, we found no relevant factor predicting better tofacitinib retention.

#### 3.3.3. Tofacitinib discontinuation and adverse events attributable to tofacitinib

Tofacitinib was discontinued in 86 (42.2%) of 204 patients; discontinuation rates were similar for biologic-naïve and biologic-experienced groups (38.3% vs. 47.7%, respectively; P = 0.23, log-rank). Treatment failure (primary and secondary) was the most common cause of discontinuation and seen at similar rates among biologic-naïve and biologic-experienced groups (primary: 36.9% vs. 32.5%; secondary: 21.9 vs. 17.5%; total: 58.8% vs. 50.0%, respectively). Rates of adverse events causing treatment discontinuation were 10.8% and 20% among biologic-naïve and experienced groups, respectively, and the difference was not statistically significant (P = 0.28). These adverse events were; in the biologic-naïve group: 2 allergic skin reactions, 1 coronary artery disease, 1 gastrointestinal bleeding, 1 knee prosthesis infection; and in the biologic-experienced group: 4 allergic skin reactions, 1 urinary tract infection, 1 pneumonia, 1 leukopenia, 1 herpes zoster. The details of the therapy switch are given in Figure 1.

The most common laboratory abnormality during the treatment course was an elevation in ALT (>3xUNL: 9.7 per 100 patient-years). Leukopenia was prevalent in 2% of patients, severe leukopenia causing drug cessation was seen in 1 patient. Neutropenia was seen in 0.5% of patients and it was not severe enough to cause tofacitinib cessation. A lipid profile at the beginning of tofacitinib administration and at the last follow-up visit under tofacitinib was available for 37 patients. HDL levels were higher at the last follow-up visit compared to the beginning of the tofacitinib regimen at significant levels (P = 0.03); LDL, total cholesterol, and triglyceride levels were similar. Incidence rates for herpes zoster and other infections were 3.9 (11 patients) and 1.4 (4 patients) per 100 patient-years, respectively. All HZ cases were monophasic, and one of 11 (9.1%) patients discontinued tofacitinib. Three of 4 patients who had hospitalization-requiring infections discontinued tofacitinib. Details of adverse events that can be attributed to tofacitinib are given in Table 3. 

**Table 3:** Adverse events attributable to tofacitinib.

Adverse Events	Value		
Leukopenia (<4000 / mm3)*	5.7		
Neutropenia (<1500 / mm3) *	1.4		
ALT > 3 X UNL*	9.7		
Lipid profile (med, Q1-4) (n = 37)	Pre	Post	P
- Total cholesterol	213 (192–243)	232 (193–261)	0.10
- LDL	138 (123–156)	145 (120–167)	0.12
- HDL	57 (46–71)	64 (53–73)	0.03
- Triglyceride	129 (99–187)	136 (104–177)	0.46
Allergic reactions/rash*	3.2		
Herpes Zoster*	3.9		
Tuberculosis*	0		
HBV reactivation*	0		
Other infections*†	1.4		
Diverticulitis*	0		
Cancer*	0		

*per 100 patient-years† Requires hospitalization: 2 pneumonia, 1 knee prosthesis infection, 1 urinary tract infection

## 4. Discussion

In this study, we reported the real-life efficacy, drug retention, and safety of tofacitinib in Turkish RA patients. Low disease activity (DAS28 ≤ 3.2) was achieved in 45.6% of patients at the last follow-up visit. Being biologic-naïve and the absence of RF were independent predictors of low disease activity. At 1 year, the overall tofacitinib retention rate was 63.9%. Disease activity at the last follow-up visit and tofacitinib retention were similar in patients receiving tofacitinib as monotherapy or in combination with csDMARDs. The rate and distribution of adverse events were similar to the current literature. 

Real-life data on JAK kinase inhibitors such as tofacitinib in RA is growing. Remission and LDA with tofacitinib have been studied extensively in clinical trials [4,10,18–20]. Long-term extension (LTE) studies of phase-3 randomized clinical trials of tofacitinib make up the main body of real-life tofacitinib evidence. One of the largest LTE studies was the ORAL Sequel LTE study that included 4290 patients. In this study, the LDA (DAS28 ≤ 3.2) rate was 46.8% at the end of the 96th month [21]. In a recent study from Switzerland, low-disease activity (DAS28 ≤ 3.2) was achieved by 58.2% of 144 RA patients on tofacitinib after a 1.2-year follow-up [22]. These data are in line with our study and the efficacy of tofacitinib was also demonstrated in small observational studies [23–25]. Besides its efficacy on disease activity, it was also shown in clinical trials that tofacitinib improves the functional status of RA patients. In the present study, the mean HAQ-DI difference was 0.40, HAQ-DI decrease ≥ 0.22 was valid for 55.3% of patients. These improvements were in parallel with the LTE studies of tofacitinib [21]. We found that being biologic-naïve and the absence of rheumatoid factor were independent predictors of good response to tofacitinib after adjusting for disease duration and baseline disease activity. Previous biologic agent use had 4.5 times higher risk of nonresponse to tofacitinib in a prospective observational study from Japan including 113 RA patients [26]; a similar association was also demonstrated by different studies [22]. The effects of serologic status on the treatment outcomes have been studied in LTE studies of tofacitinib RCTs. In that analysis, response rates were higher in seropositive patients compared to seronegative patients (response rates: anti-CCP+/RF+ > anti-CCP–/RF–, anti-CCP+/RF– > anti-CCP–/RF–, anti-CCP–/RF+ > anti-CCP–/RF–) [27]. However, this association has not been fully confirmed by real-life data. Similar to our study, there was no difference in anti-CCP positivity among responders and nonresponders in studies by Mori, Iwamoto, Mueller, and colleagues. Mori et al. did not report RF status; the others reported no relationship between RF status and response rates [19,22,26]. Also, there are conflicting data regarding the RF status and response to TNF inhibitors. Some of these studies suggested that RF positivity is a risk factor for poor response to TNF inhibitors [28–32]. Data regarding the link between serologic status and response to tofacitinib is scant and conflicting. Further studies are needed to enlighten the mechanism and clinical application of this link. 

Overall tofacitinib retention rate at 1 year was 63.9%. This rate was similar across other tofacitinib real-life data [22,25], however, slightly lower than the retention rate of anti-TNF agents [33]. For tofacitinib, being a late player in the field of RA may be an explanation of this reduced retention. However, when anti-TNF agents were compared with tofacitinib when all the treatments were started in the same time period, retention rates were similar. Even higher for tofacitinib when the adjustments for potential confounders were done [25]. We found no predictor of better tofacitinib retention, including the status of previous biologic DMARD use and using tofacitinib as monotherapy or in combination with csDMARDs. However, real-life data from Israel reported an inverse relationship between the number of previous bDMARDs and tofacitinib retention similar to anti-TNF agents and they found no other relevant factors [34,35]. 

We found similar response and retention rates of tofacitinib in patients with and without concomitant csDMARDs. There are many studies demonstrating the efficacy and safety of tofacitinib monotherapy. The ORAL Solo trial showed the efficacy of tofacitinib monotherapy in reducing RA signs and symptoms and improving physical function in patients with inadequate response to disease-modifying drugs [5]. In ORAL Standard trial, tofacitinib add-on to methotrexate was superior to adalimumab add-on to methotrexate therapy [8]. The results of ORAL Strategy trial for tofacitinib monotherapy were defined as statistically inconclusive because noninferiority of tofacitinib 5 mg b.i.d. to either adalimumab and MTX or tofacitinib and MTX was not shown [10]. Concomitant csDMARDs were found to be required for optimal treatment results for TNFi but not for tofacitinib and non-TNFi in SCQM cohort [25]. A systematic review and metaanalyses showed that tofacitinib monotherapy was neither statistically nor clinically different from TNF inhibitors in efficacy [36,37]. In addition, we noticed that our csDMARD strategies were different from the literature. Leflunomide (LEF) and hydroxychloroquine (HCQ) utilization rates were higher in the present data. For instance, Mueller and colleagues reported LEF and HCQ rates were 17.3% and 7.6%, respectively, in their cohort [22]. Also, participants of ORAL Solo trial were allowed to use HCQ, and the rate was 18.5% [5]. Although the cohort was relatively small to conclude it, LEF seems an important player just behind MTX. Also, further assessments are needed if there was a possible cardiovascular protective contribution of HCQ to neutralize cardiovascular adverse effects. Prospective, large-scale studies are needed to reveal these important points.

A total of 23% of our patients discontinued tofacitinib due to ineffectiveness. Clinical trials or their LTE studies did not report clearly on this issue. Recent real-life data reported the drug discontinuation rate due to ineffectiveness as 15.9% [22]. This rate is a bit lower than ours, however, the differences between these two cohorts regarding demographics, disease, and treatment characteristics may explain this discrepancy.

The safety profile, including infections and laboratory anomalies, of our cohort, is consistent with the current literature [21,22,38,39]. We had no HBV reactivation and tuberculosis, which may be due to the strict surveillance and prophylaxis regimen of Turkey. None of the patients had a cancer diagnosis under tofacitinib, however, the follow-up duration was not enough to make a decision. Herpes zoster was the most common infection in our cohort. We found an HZ incidence rate similar to that reported from the USA and global data; however, we found a lower incidence rate than reported from far East Asia [38]. Most patients had HZ in only one dermatome (92%), and 8% of patients with HZ discontinued tofacitinib permanently, similar to the current study [21,40]. 

The main limitation of our study was its one-center design. Our results should be validated in larger and multicenter studies. We could not test the cardiovascular risk of tofacitinib properly. Besides, we did not examine the effect of tofacitinib on radiographic progression. Also, as we could not clearly assess the drug compliance of the patients (e.g., we accepted the patients with control visit within 6 months in “tofacitinib-continue group” but the patients might step-down to csDMARDs without informing the treating physician, or patients might use the drug irregularly) and these issues may cause under/overestimation of drug retention. However, a 3-month regular follow-up regulation of our social security system has minimized the bias caused by the 6-month cut-off.

In conclusion, tofacitinib is effective as monotherapy or in combination with csDMARDs and is a well-tolerated treatment option in Turkish RA patients. The safety profile is consistent with current literature. 

**Conflict of interest**

LK, OK, SAB, SK, AIE, and UK received consultancy fees and/or speaker fees from Abbvie, Amgen, Janssen, Novartis, Pfizer, Roche, UCB Pharma. Other authors have no conflict of interest to report.

**Informed consent**

Our study is compliant with the Helsinki Declaration and approved by Hacettepe University ethical committee (approval number: GO 19/1088).
